# Do We Need Radiotherapy in Grade II Ependymoma?

**DOI:** 10.3389/fonc.2022.800505

**Published:** 2022-03-04

**Authors:** Aleksandra Napieralska, Wojciech Majewski, Leszek Miszczyk

**Affiliations:** Radiotherapy Department, Maria Sklodowska-Curie National Research Institute of Oncology Gliwice Branch, Gliwice, Poland

**Keywords:** ependymoma, brain tumor, adjuvant treatment, radiotherapy, neuro-oncology

## Abstract

**Purpose/Objectives:**

The debate on whether radiotherapy (RT) is an essential part of primary treatment in patients with grade II ependymoma (G2E) is still ongoing, and this study aimed to evaluate its role.

**Materials/Methods:**

A retrospective analysis of all the consecutive patients treated due to G2E in years 1985–2019 was performed. The group consisted of 116 patients with a small predominance of woman (55% vs. 45%) and the location of the tumor in the brain (58% vs. 42%). All had surgery as the primary treatment with 47% R0 resection. Radical RT was applied in 81 patients. In majority of cases (91%), patients received local irradiation.

**Results:**

Median follow-up was 65 months, and during that time, 17 patients died. Five- and 10-year overall survival (OS) of the whole group was 87% and 83%. Radical surgery (R0 vs. R1/2) improved OS (*p* = 0.004), but the difference was observed only in patients with brain lesions (*p* = 0.01). Five- and 10-year progression-free survival (PFS) was 68% and 51%, respectively. Looking at the treatment of recurrence, those who received RT as a part of the treatment of the recurrent tumor had better OS (*p* = 0.048)—5- and 10-year OS of 85% and 78% vs. 66% and 57%. In the multivariate analysis, radical surgery (R0 vs. R1/2) and the use of RT in the primary treatment improved PFS (*p* = 0.006 and 0.007). Based on the location of the tumor, the positive influence of RT on PFS was observed only in the case of patients with brain tumors (*p* = 0.01). Also, comparing R1/2 surgery with R0 resection—the benefit of RT was only observed in R1/2 group (0.02).

**Conclusions:**

RT in the case of patients with G2E is a valuable treatment of the recurrent disease. Patients with brain lesions after nonradical surgery might benefit from the local irradiation in terms of PFS.

## Introduction

Ependymomas are rare central nervous system tumors and represent less than 2% of central nervous system tumors in adults and less than 6% in children (1). The Current National Comprehensive Cancer Network (NCCN) and The European Association of Neuro-Oncology (EANO) guidelines recommend maximal safe resection with adjuvant radiotherapy (RT) or observation for patients with G2 ependymomas (G2E). Local irradiation is advised in case of brain lesions or subtotal resection of spinal tumors (2, 3). Nevertheless, since the published studies concerning the role of RT are inconclusive, further analysis is needed (4–43). The present study was undertaken to define the long-term outcome of patients with G2E in the last 35 years. Treatment options and prognostic factors were evaluated to clarify their relationship to survival and disease control and to work-out treatment recommendations. An additional systematic review of published studies of patients with spinal and cranial G2E was performed.

## Material and Methods

A retrospective analysis of patients treated in years 1985–2019 due to ependymoma was performed. Study inclusion criteria were histopathologically confirmed G2E, at least 2 months of follow-up from the date of the diagnosis and an available data concerning delivered radical RT. In all the cases, the diagnosis was based on the radiologic imaging (computed tomography (CT) in earlier years of the study and magnetic resonance (MR) evaluation of the brain or spine) and pathologic examination of the tumor tissue samples. The diagnosis of other brain tumor in the past was not an exclusion criterion if the pathologic examination confirmed different histopathology of a tumor and no brain irradiation had been performed. The study was approved by the hospital ethical committee and performed in accordance with the Helsinki Declaration.

The survival and local control rates were the primary endpoints of the analysis. Overall survival (OS) was calculated from the date of the diagnosis of the tumor to the date of death or the date of the last follow-up visit. Progression-free survival (PFS) was calculated from the date of the diagnosis of the tumor to the date of documented progression (local or distant—based on diagnostic imaging) of the tumor or the date of death of the patient. Eastern Cooperative Oncology Group (ECOG) scale was used to classify patients’ performance status. In statistical analysis, OS and PFS were calculated using Kaplan–Meier method. The missing dates of deaths were obtained from the Polish National Cancer Registry. Patient’s outcomes were divided into 4 categories: alive with no evidence of disease (NED); alive with disease (AWD); dead of concurrent/intercurrent disease (DOC); and dead of unknown cause (DUC). Median follow-up was estimated by the Kaplan–Meier analysis with the reversed meaning of the status indicator. Various patients’ characteristics were included in the univariate and multivariate analyses to identify their impact on OS and PFS. The comparisons were made with the use of the log-rank test and Cox-regression model. Variables with *p*-value of <0.05 in the log-rank test and univariate Cox analysis were used in the multivariate Cox analysis. The comparison of characteristics of patients in RT and no-RT group was performed with Student’s *t*- and Chi-2 tests. A *p*-value of ≤0.05 was regarded as being statistically significant. Statistical analysis was performed using Statistica software (version 12.0).

The literature review was performed using PubMed and included all articles published from 1996 to 2020. Terms used were as follows: “ependymoma”, “radiotherapy”, “grade 2 tumors”, and “grade II ependymoma”. The manual searches of the reference lists of the included studies were used to identify the ones that the electronic search might have failed to identify. Studies included must meet the inclusion criteria: the subtype of G2E according to the WHO classification, the number of patients with G2 tumors if more than one subtype was the topic of the article and the follow-up and information about the outcome, at least part of the patients must have received radiotherapy as part of primary treatment, the article must be published in English, and full text of the article had to be available.

## Results

### Patient’s Characteristics

During the study period, 244 patients with ependymoma were identified in the hospital database and 116 had histopathological diagnosis of G2E and met study inclusion criteria. Two independent pathological evaluations of tissue samples were conducted in 43 patients (37%). Among others: 19 (16%) had specimens evaluated in medical university pathology department, 9 (8%) had evaluation in forensic department, 6 (5%) in neuropathology department, and 6 (5%) more had evaluation carried in neurosurgical hospital outside our region. Remaining 29% had tissues evaluated in pathology departments, mainly in our region, but the name of the institution which performed the evaluation was unavailable. Information about Ki67 index was available in 27 patients, and it ranged from 1% to 100% (median 1%). The patients’ and treatment characteristics are presented in **Table 1**. The comparison of RT and no-RT group is presented in **Table 2**. The most common symptoms in patients with cranial tumors were local pain (61%), visual deficits/dizziness (42%), vomiting/nausea (36%), and consciousness disturbances/faint (24%). Frequently reported symptoms among those with spinal lesions were local pain (71%), paresis (35%), and paresthesia (29%). The diagnosis was based on imaging—MR with or without CT was performed in 80% of the patients and 20% had solely CT.

**Table 1:** Patients’ and treatment characteristics.

Characteristic	
Age at diagnosis	*Value*
Median	35
Range (SD)	2–82 ( ± 17.9)
Children	15 (13%)
Adults	101 (87%)
Sex	*Number of patients*
Female	64 (55%)
Male	52 (45%)
ECOG performance status	
0	29 (25%)
1	76 (66%)
2	8 (7%)
No data	3 (2%)
Primary site	
Brain	67 (58%)
Supratentorial	22 (19%)
Infratentorial	45 (39%)
Spinal cord	49 (42%)
Dissemination at diagnosis	
Yes	9 (8%)
No	107 (92%)
Underwent surgery	
Yes	116 (100%)
Total (R0)	55 (47%)
Subtotal (R1 or R2/biopsy)	55 (47%)
No data	6 (6%)
Underwent radiotherapy in primary treatment	
Yes	81 (70%)
No	35 (30%)
Underwent chemotherapy in primary treatment	
Yes	6 (6%)
No	110 (94%)

ECOG, Eastern Cooperative Oncology Group; R0, radical resection (macro- and microscopically); R1, macroscopically radical resection (but not microscopically); R2, macroscopically nonradical resection; SD, standard deviation.

**Table 2:** Patients’ characteristic in radiotherapy (RT) and no-radiotherapy (no-RT) group.

Characteristic	RT	No-RT	*p*-value
Age (mean)	36.3	36.4	0.977
Gender			0.594
Male	43%	49%	
Female	57%	51%	
ECOG performance status			0.320
0	28%	21%	
1	63%	76%	
2	9%	3%	
Primary site			0.748
Brain	57%	60%	
Spine	43%	40%	
Dissemination at diagnosis			0.331
Yes	6%	11%	
No	94%	89%	
Surgery			0.147
R0	49%	52%	
R1/R2	51%	48%	
MRI in diagnosis			0.168
Yes	83%	71%	
No	17%	29%	

ECOG, Eastern Cooperative Oncology Group; MRI, magnetic resonance imaging; R0, radical resection (macro- and microscopically); R1, macroscopically radical resection (but not microscopically); R2, macroscopically nonradical resection.

### Primary Treatment—Surgery

Surgery was the primary treatment modality in all of the patients. Mean preoperative tumor dimensions were 3.9 × 2.5 × 2.3 cm (SD ± 2.7 × 1.6 × 1.3 cm). The extent of the resection (EOR) was determined by radiologist based on the postoperative imaging (CT or MR performed 24 to 48 h after the surgery). However, operative notes were also used to assess EOR, especially if imaging was not available. EOR was designated as either a subtotal resection [STR, which included biopsy only, gross residual determined by a neurosurgeon, or residual enhancement on postoperative imaging, meaning macroscopically but not microscopically radical resection (R1) and macroscopically not-radical resection (R2)] or gross total resection [GTR, defined as a complete resection determined by the neurosurgeon and/or radiologist, meaning macro- and microscopically radical resection of the tumor (R0)]. Fifty-five patients (47%) had GTR confirmed with MR. The second and third surgeries were performed in 14 and 2 patients, respectively. During the next surgery, a higher-grade (G3) tumors were found in 4 patients (in 3 children and 1 adult).

Chemotherapy (CTH) was part of the primary treatment in 6 cases. All patients who received CTH were children and in 3 of them anaplastic ependymoma was found during the second surgery. In Poland, majority of pediatric oncologic centers apply CTH according to the national protocols and all children included into the study were treated in accordance with the same national protocol. The systemic agents used were as follows: etoposide 150 mg/m^2^ (days 1–3 and 42–44) and ifosfamide 3 g/m^2^ (days 1–3, 21–23, 42–44, and 63–65). All children received 4 cycles of CTH after the surgery and before the adjuvant RT. Two of them were previously diagnosed with other brain tumor: one with xanthoastrocytoma 3 years earlier and the other one with a meningioma 1 year earlier. Among the adults, one patient was a survivor of breast cancer and one was diagnosed with neurofibromatosis type 2 and had malignant peripheral nerve sheath tumor of the optic nerve. During the follow-up, one patient was diagnosed with skin melanoma and died due to the progression of this disease. Two more patients were diagnosed with asymptomatic meningiomas within the irradiation field.

### Primary Treatment—Radiotherapy

**Figure 1:**
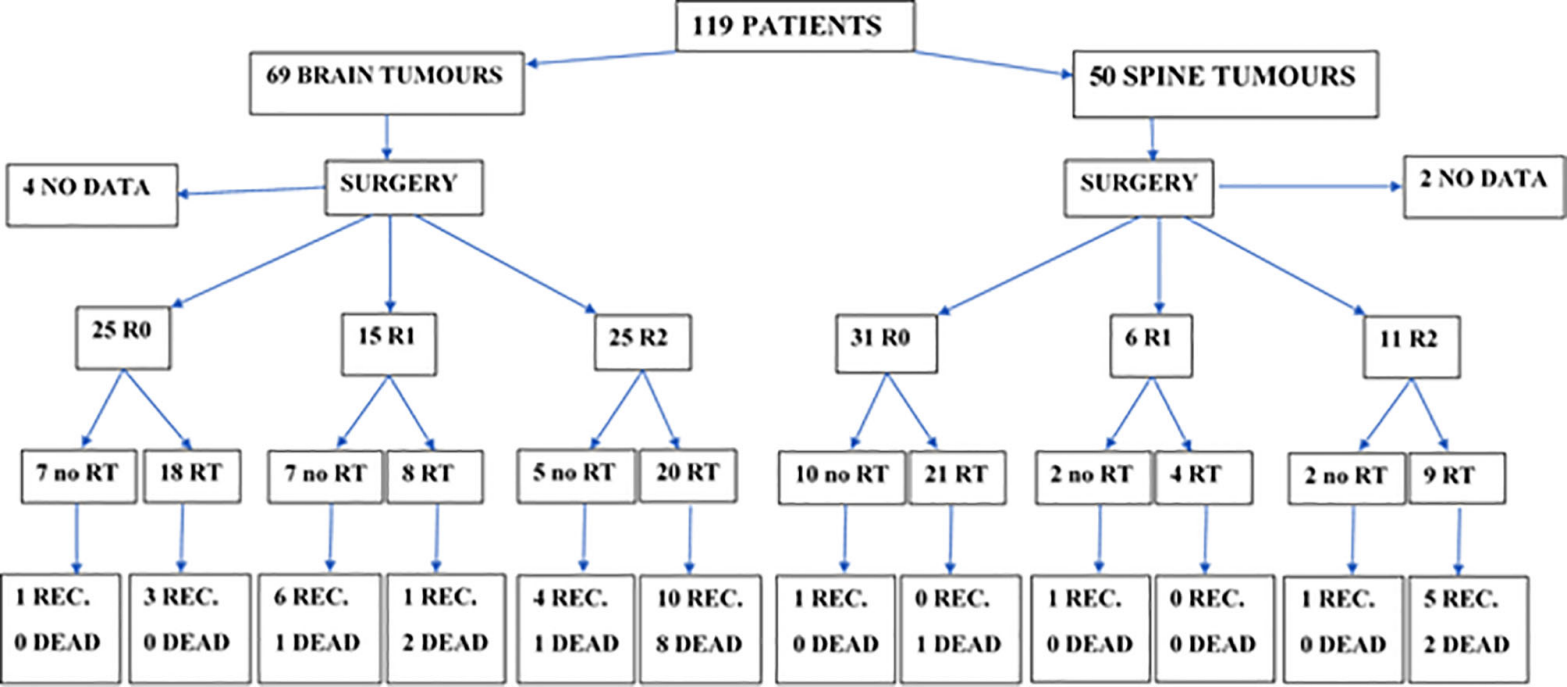
Primary treatment and treatment results in patients with brain and spinal tumors (CTH not included, used in the treatment of 5 patients with brain tumors after R2 resection in 4 of them and R1 in one, and in one patient with spinal tumor and lacking data about the extent of resection; all of them received RT in primary treatment). REC, recurrence; RT, radiotherapy; R0, radical resection (macro- and microscopically); R1, macroscopically radical resection (but not microscopically); R2, macroscopically not-radical resection.

Radical RT was part of the primary treatment in 81 cases (70%). In 8 patients, information regarding irradiation technique was unavailable. Among the other 73 patients, 9 were treated with old radiotherapy techniques (2D/Cobalt), 17 had 3D conformal radiotherapy (3D CRT), and 47 was irradiated with new techniques—19 with VMAT, 26 with IMRT, and 2 with TomoTherapy. A thermoplastic mask was used for treatment position reproducibility. The clinical target volume (CTV) and normal structures have been defined on CT with MRI fusion for the patients treated since introduction of Eclipse planning system in year 2003. Among those who were irradiated with 3D CRT or dynamic techniques, 13 had treatment planning based only on CT and in 51 patients MRI was used to improve delineation process. The gross tumor volume (GTV) included the operative bed and the residual tumor as defined by contrast-enhanced T1-weighted, T2-weighted, and fluid-attenuated inversion recovery (FLAIR) sequences. In pre-MR era, preoperative and postoperative contrast-enhanced tumor visible on CT was considered GTV. Seventy-two patients had local irradiation and the CTV covered tumor bed in 46 patients, residual tumor with tumor bed in 19 patients, ventricular system with tumor bed in 2 patients, and part of involved spinal canal with additional margin in 5 patients. An additional geometric expansion (usually 5–7 mm) was added to the abovementioned CTV to create the planning target volume (PTV) to account for setup errors and intrafraction motion. CranioSpinal Irradiation (CSI) was applied in 7 patients. Characteristics of the doses and fields used in primary treatment are presented in **Table 3**. In two patients, data concerning the field and fractionation were not available due to the changes in the treatment planning system. RT was delivered with protons in two patients and with the use of 6–23 MV photon beams in all the others. In the case of patients who received CSI, the total dose (TD) of 30.6–36 Gy (median 36) was delivered to craniospinal axis. One patient did not completed RT due to the deterioration of performance status and finished the irradiation after the total dose of 43.2 Gy. Primary treatment applied in all of the patients is presented on **Figure 1**.

**Table 3:** Radiotherapy characteristics.

Group	Number of pts	TD (range) (Gy)	Median TD (Gy)	fd (range) (Gy)	Median fd (Gy)	Local irradiation/craniospinal/ND
**Primary treatment**						
Brain tumors	46	45.0–60.0	54.0	1.6–2.0	1.8	40/5/–
Spinal tumors	35	41.4–56.0	45.0	1.6–2.0	1.8	32/2/–
**Recurrence treatment**						
Brain tumors	17	7.0–54.0	18.0	1.5–18.0	5.5	11/6/–
Spinal tumors	3	30.0–45.0	37.5	1.8–1.9	1.8	2/0/1

fd, fraction dose; Gy, grey; ND, no data; pts, patients; RT, radiotherapy; TD, total dose.

### Recurrence Treatment

The recurrence of the disease was observed in 34 patients (29%) after median time of 61 months (range 1–407 months). All but 7 patients received some form of local treatment (surgery or RT) of recurrent lesions. Characteristics of RT applied in recurrence treatment are presented in **Table 3**. The effect of this RT was evaluated in 13 cases with stagnation of the tumor observed in 12 of them and partial regression in one. Total number of patients who received second and third courses of RT was 13 and 1, respectively. Metastases were diagnosed in 8 patients during the follow-up with spinal canal as the most common location of the dissemination (in 6 of them).

### Overall Survival

Median follow-up was 65 months, and during that time, 17 patients died. Five- and 10-year OS of the whole group was 87% and 83%. Patients with brain lesions had 5- and 10-year OS of 84% and 77% compared with 5- and 10-year OS of 93% and 93% for patients with spinal tumors. At the end of follow-up, 10 patients are DOC, 7 DUC, 31 AWD, and 68 NED. Patients younger than 30 years old (*p* = 0.08) and those with the spinal location of the tumor (*p* = 0.08) tended to live longer. Only GTR improved OS (*p* = 0.004) with 5- and 10-year OS of 97% and 97% vs. 81% and 74%, respectively, for GTR vs. STR. Looking at the location of the tumor, the difference between GTR and STR was observed only in patients with brain lesions (*p* = 0.01) with no difference in patients with spinal tumors (*p* = 0.51). Patients with cranial tumors and R0 resection had 10-year OS of 100% compared with 10-year OS of 68% for patients with no radical surgery. CTH or RT did not influence survival of the whole group, and what is more, RT, applied in the case of STR, did not improved OS. What is interesting is that patients who presented with pain had better OS compared with those with no such primary symptom of the disease (*p* = 0.02). Looking at the treatment of recurrence, those who received RT as part of the treatment of the recurrent tumor had better OS (*p* = 0.048)—5- and 10- year OS of 85% and 78% vs. 66% and 57% for patients without irradiation in recurrence treatment, respectively.

### Progression-Free Survival

**Figure 2:**
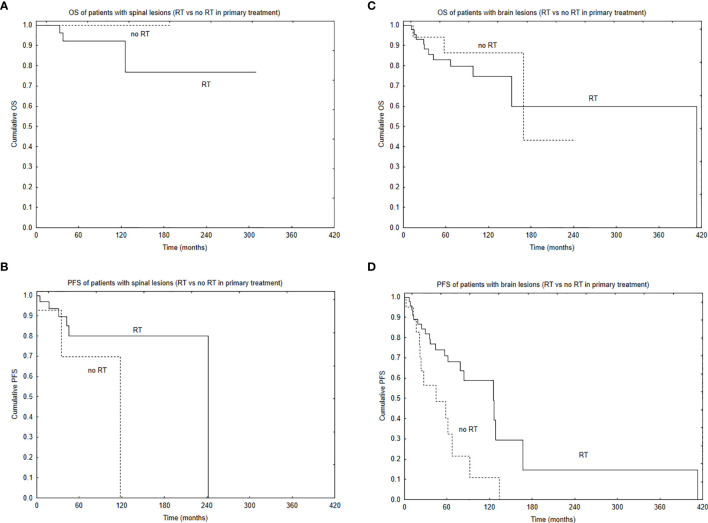
Survival curves based on the use of radiotherapy (RT) in primary treatment with the regard to tumor location: overall survival **(A)** and progression-free survival **(B)** of patients with spinal location of the tumor based on the use of RT in primary treatment and overall survival **(C)** and progression-free survival **(D)** of patients with brain location of the tumor based on the use of RT in primary treatment.

**Figure 3:**
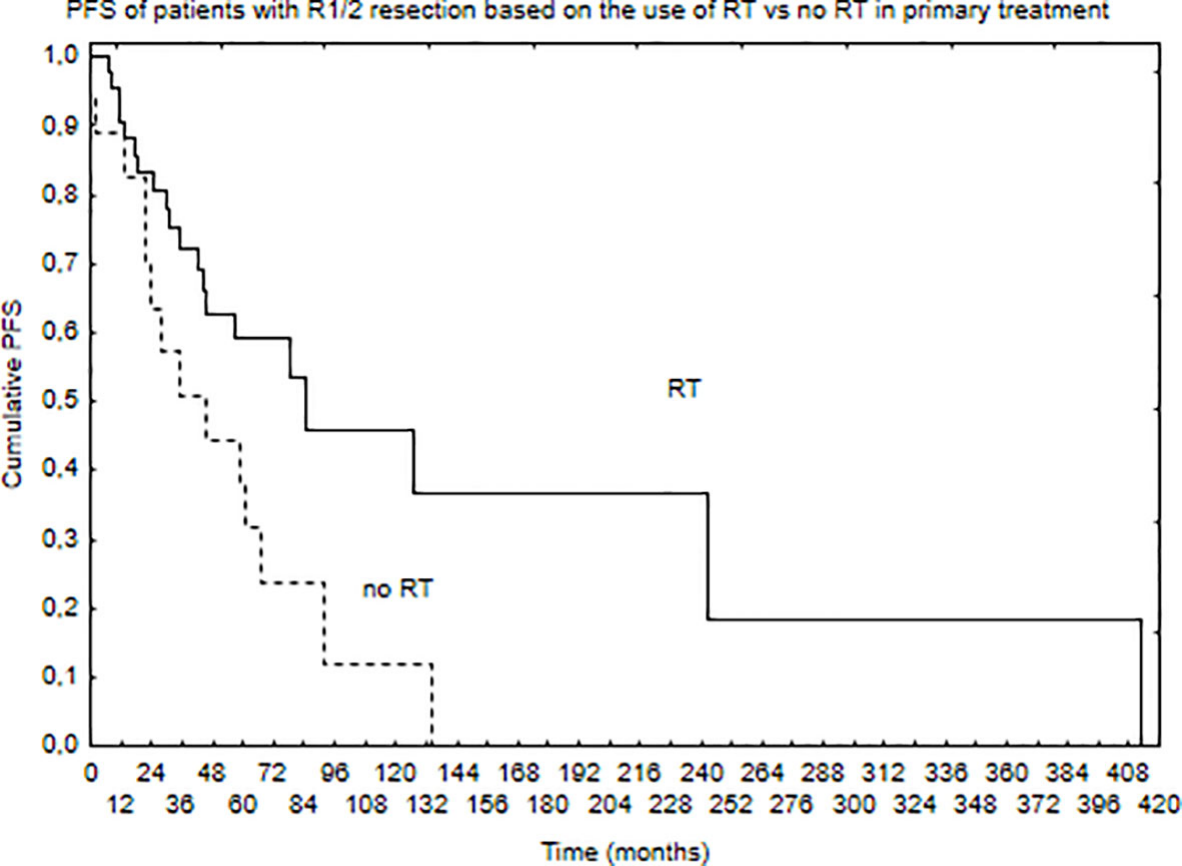
Progression-free survival of patients with STR (R1 or R2 resection) of the tumor based on the use of RT in primary treatment (*p* = 0.02).

**Figure 4:**
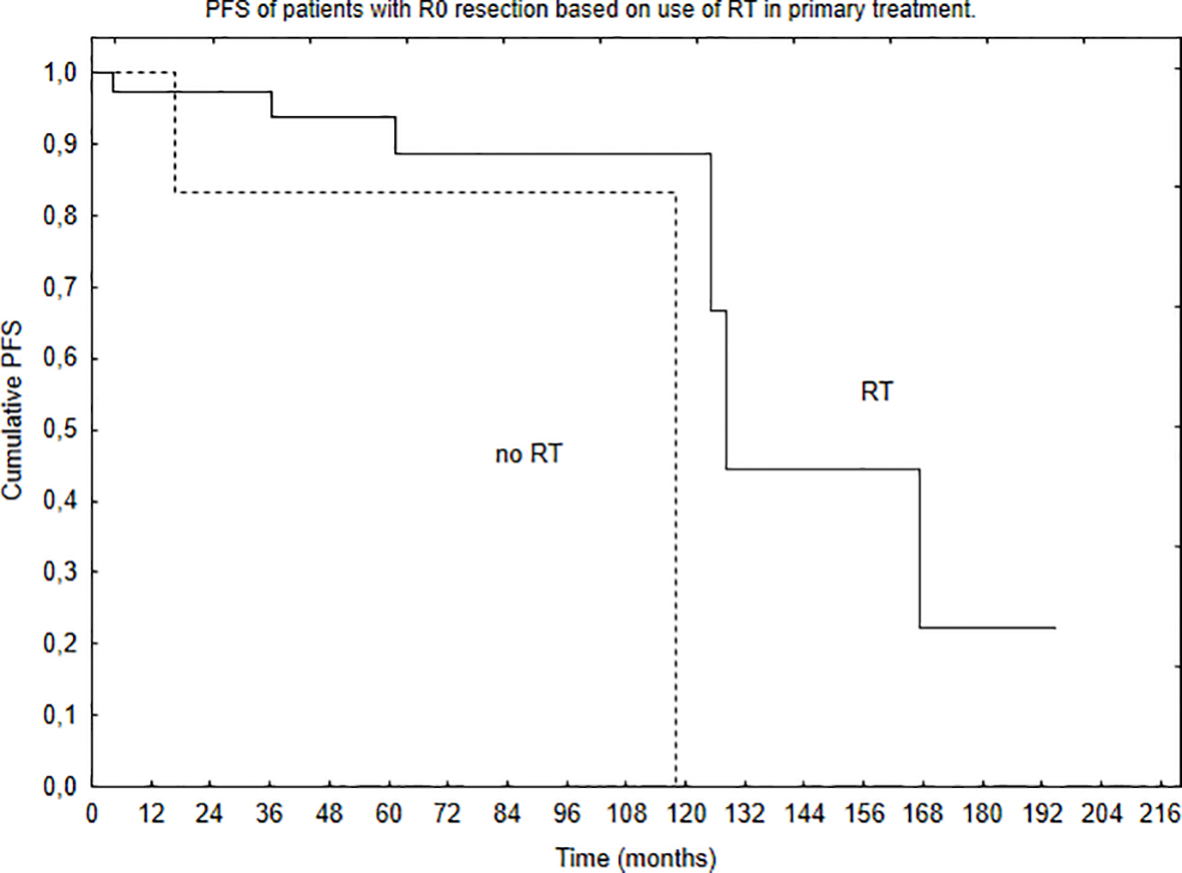
Progression-free survival of patients with GTR (R0 resection) of the tumor based on the use of RT in primary treatment (*p* = 0.29).

Median PFS was 65 months, and during that time, local, local and distant, and distant recurrence was diagnosed in 34, 7, and 4 patients, respectively. Five- and 10-year PFS of the whole group was 68% and 51%. Patients with brain lesions had 5- and 10-year PFS of 63% and 45% compared with 5- and 10-year PFS of 78% and 62% for patients with spinal tumors. The univariate analysis confirmed that results with *p* = 0.025. EOR (GTR vs. STR, *p* = 0.001) and use of RT in the primary treatment (*p* = 0.003) had positive influence on PFS in the univariate analysis. The presence of nausea/vomiting had negative impact on PFS (*p* = 0.03). Also, patients with brain lesions and presence of paresis had worse PFS (*p* = 0.03). The multivariate analysis confirmed the impact of EOR and RT on improved PFS (*p* = 0.006 and 0.007). Positive influence of RT on PFS was observed only in the case of patients with brain tumors (*p* = 0.01, **Figure 2**); however, the number of events was very small in patients with spinal tumors. Also, comparing those who had STR with GTR—the benefit of RT was observed in the STR group (*p* = 0.02, **Figures 3** and **4**).

## Discussion

According to the WHO 2016 classification, central nervous system ependymal tumors can be classified as subependymomas (WHO G1), myxopapillary ependymomas (WHO G1), ependymomas (WHO G2), ependymomas RELA fusion-positive (WHO G2 or G3), or anaplastic ependymomas (WHO G3) (44). Treatment of ependymomas is one of the most controversial issues in neurooncology. EANO guidelines, based on the current WHO classification divided patients into nine subgroups based on anatomical location (supratentorial, posterior fossa, spinal) and patient age. Distinct genetic and epigenetic alterations had been identified, and distinct outcomes for each group were reported. Such findings may lead to more a precise diagnostic and prognostic evaluation, therapies dedicated to molecular subgroup (like the one led by Merchant TE), and in the future, new recommendations (3, 26). As a consequence of very low incidence of adult ependymomas, there is a lack of prospective clinical trials in this population and most of the reported series on these neoplasms are retrospective or include limited number of patients (3, 5, 7, 10–43, 45) (**Tables 3** and **4**). Only several prospective studies were conducted in the pediatric population which all concluded that RT should be part of the treatment (26).

**Table 4:** Studies of patients with cranial grade II ependymomas with regard on the impact of adjuvant radiotherapy.

In favor of adjuvant radiotherapy	Against adjuvant radiotherapy
Reni M et al. (42) 51 adults: 10-year OS: 59% vs. 68%—RT vs. no RT; 10-year FFS: 34% vs. 12%—RT vs. no RT	Dutzmann et al. (10) 64 adults (33 G II): 10-year OS: 90% vs. 82%—RT vs. no RT
Metellus et al. (6) 114 adults: 10-year OS: 87% vs. 78%—RT vs. no RT	Aizer et al. (11) 112 adults, SEER database: not reported OS/PFS with respect to RT
Snider et al. (12) 482 children ≤3-year olds (302 G II); SEER database: 10-year OS: 50% vs. 43%—RT vs. no RT	Nuno et al. (13) 1,318 pts (1,055 G II); NCDB: 10-year OS: 63% vs. 70%—GTR + RT vs. GTR; 10-year OS: 70% vs. 70%—STR + RT vs. STR
Guyotat et al. (14) 106 adults (88 G II): 10-year OS: 86% vs. 77%—RT vs. no RT; 10-year PFS: 75% vs. 50%—RT vs. no RT	Roldan Urgoiti et al. (15) 139 pts (106 brain and spine G II): not reported OS/PFS with respect to RT
Metellus et al. (16) 152 adults (109 G II): 10-year PFS: 75% vs. 45%—RT vs. no RT	Wang et al. (17) 55 patients (17 G II); supratentorial extraventricular tumors: not reported OS/PFS with respect to RT
Rodriguez et al. (18) 2,408 pts (2,132 spine and brain G II); SEER database: not reported OS/PFS with respect to RT	Prabhu et al. (4) 1,787 adults (1,471 G II); NCDB: 5-year OS: 82% vs. 88%—RT vs. no RT
Woo Wee et al. (19) 172 adults (106 G II): 10-year PFS and OS: 54% and 82% vs. 41% and 80%—RT vs. no RT	Korshunov et al. (20) 258 pts (131 low G): 5-year OS: 95% vs. 95%—RT vs. no RT
Ernestus et al. (21) 67 children (38 G II): not reported OS/PFS with respect to RT	Wang et al. (22) 30 pts (17 G II) + literature review of 106 pts (68 G II): 10-year OS and PFS: 95% and 80%—RT and no RT
Cage et al. (23) literature review; 182 children (115 GII): 5-year OS: 87% vs. 73%—GTR + RT vs. GTR	Ailon et al. (24) 26 children (23 GII): 10-year OS and PFS: 65% and 60% vs. 72% and 58%—RT vs. no RT (posterior fossa tumors); 10-year OS and PFS: 67% and 58% vs. 44% and 40%—RT vs. no RT (supratentorial tumors)
Perilongo et al. (25): 92 children (61 GII): 10-year OS and PFS: 58% and 41% vs. 52% and 20%—RT vs no RT (whole group)	Vera-Bolanos et al. (43) 282 adults (212 spine and brain G II): 10-year PFS: 75% vs. 58%—GTR vs. GTR + RT; 10-year PFS: 58% vs. 50%—STR vs. STR + RT
Merchant et al. (26) 356 children (215 GII), ACNS0121 study: 10-year EFS and OS: 69% and 82% vs. 33% and 49%—RT vs. delayed/no RT	Amirian et al. (46) 367 pts (348 GII spine and brain); SEER analysis: not reported OS/PFS with respect to RT
Rogers L et al. (27): 45 pts (43 low G): 5-year LC: 100% vs. 75%—RT vs. no RT; 10-year OS: 67% vs. 43%—GTR + RT vs. GTR	
Pejavar S et al. (28) 39 children (19 G II): 10-year PFS and OS: 36% and 68% vs. 0% and 61%—RT vs. no RT (whole group)	
Ernestus et al. (29) 126 pts (87 G II): 5-year PFS: 76% vs. 48%—RT vs. no RT	
Deng et al. (30) 632 children: 5-year OS and CSS: 71% vs. 62% and 73% vs. 67%—RT vs. no RT	

On the left studies in favor of adjuvant radiotherapy, on the right against postoperative irradiation (4, 6, 10–30, 42, 43, 46).

CSS, cancer-specific survival; EFS, event-free survival; FFS, failure-free survival; G, grade; GTR, gross total resection; LC, local control; NCDB, National Cancer Database; NTR, near-total resection; OS, overall survival; PFS, progression-free survival; pts, patients; RT, radiotherapy; SEER, the Surveillance, Epidemiology and End Results; STR, subtotal resection; vs., versus.

### Cranial Ependymomas

Adult ependymomas are rare tumors that still generate controversy with regard to their clinical management (2, 3, 6, 47). Surgery plays an important role, and many authors reported that EOR impacts the outcome. However, GTR can be hindered by anatomical factors, such as adherence of the tumor to the nearby organs. Reoperation following initial incomplete surgery or at tumor recurrence is advocated, assuming that GTR could be achievable. However, surgery alone especially in the case of STR, is rarely curative and the vast majority of relapses are local (6, 11, 12, 14–20, 22, 23, 25–27, 30, 42, 43, 45, 46). What is interesting is that a few authors have found no correlation between the EOR and the prognosis, suggesting that in some cases, less-aggressive approach combined with an adjuvant irradiation can provide similar outcome as GTR (10, 27, 28). The positive influence of EOR on survival was observed in our study, and those who had GTR of the tumor presented the best outcome (**Figure 2**). Radical resection, confirmed with postoperative MR is advocated for all patients suitable for such surgery.

Among other factors found to positively influence OS were younger age with cutoff value within the range of 30–55 years and such correlation was also observed in our study (4, 6, 10, 13, 16, 27, 42, 46). Only Vera-Bolanos et al. reported that patients younger than 44 years old had worse PFS compared with the older ones, but in her study, spinal and cranial tumors were mixed, and patients with tumors of other than G2 were included (43). Another factor identified in many studies as associated with worse survival prognosis was the supratentorial location of the lesion (6, 10, 13, 15, 20, 24, 43, 46). In contrast, Aizer et al. found that patients with supratentorial tumors are having the better outcome. Based on their analysis and literature review of studies delaying RT in the case of completely resected supratentorial tumors, they suggested that irradiation could be reserved in case of recurrent lesions with part of the patients avoiding radiation-induced brain toxicity (11, 16, 24). Their observations regarding the location impact were not confirmed by any other author. We did not find the correlation between the supra- and infratentorial tumors and survival in our study.

The role of RT is one of the most debatable issues in terms of G2E treatment. The influence of RT in case of GTR in cranial tumors was not confirmed by several authors (4, 10, 13–15, 18, 24). In our study, no benefit from RT after GTR in terms of OS or PFS was observed, although vast majority of those patients received irradiation. There is a possibility that delaying RT in such patient population could be considered in highly selected cases. However, the absolute benefit of adjuvant RT in adult group has not been validated *via* prospective randomized trial, and there is no ongoing trial as well, so its value can only be examined based on the available retrospective data to date (26). Our analysis of patients after STR showed that this group could benefit from postoperative RT in terms of PFS which was also observed by other authors (6, 14, 16, 18, 19, 47–49). This thesis, to administer adjuvant RT to patients after STR of intracranial G2E is also supported by EANO and NCCN consensus guidelines (2, 3). However, there is no concrete evidence as to which therapeutic strategy is optimal, especially with regard to the time point and extent or RT (48). Concepts regarding target volume for RT changed over the years. In the past, patients with ependymoma often received whole brain RT or CSI. Since several studies demonstrated that local irradiation is sufficient to achieve good results, the treatment fields shrank to cover only the local extent of the disease (tumor/tumor bed with additional margin of 1–2 cm), so with no evidence of dissemination, local RT to total dose of 54–59.4 Gy is advocated (3, 26, 48, 49). The CSI is reserved for cases with dissemination diagnosed based on MR or the cerebrospinal fluid analysis. There is also a possibility to consider a boost to tumor bed and any gross disease after the dose of 54 Gy in cases with lesions located in a close proximity of organs at risk (49).

The role of CTH either before or following RT remains uncertain. Recurrent ependymoma remains a challenge without a uniformly accepted approach. Some patients undergo an additional course of RT, which as shown in our study, could provide better survival (3, 49). Conventional or hypofractionated stereotactic irradiation photon therapy as well as protons could provide satisfactory local results (3). Whenever possible, repeated surgery is also advocated (2, 3). Long-term follow-up with MR is recommended for all the patients, regardless of the EOR.

### Spinal Ependymomas

Patients with spinal cord ependymomas have better prognosis than with other intramedullary glial tumors or brain-located ependymomas (43, 46, 49). However, factors affecting prognosis have not been clearly defined for this population and simple extrapolation from studies on intracranial ependymomas is not valid (31, 34). The management of spinal ependymomas typically begins with surgery, which offers removal of mass effect and can achieve oncologic control in some patients (3, 33). The postoperative MR confirming GTR is strongly recommended. Similarly to patients with brain lesions, EOR was also found as the most important factor influencing survival (3, 8, 31–35, 40, 41, 43, 46) (**Table 5**). The goal of GTR must be balanced against the risk of aggressive surgery, which depending on the lesion location can cause severe neurologic deficits. Complications following spinal tumor resection include motor and sensory loss, bowel and bladder dysfunction, and cerebrospinal fluid leaks (34).

**Table 5:** Studies of patients with spinal grade II ependymomas with regard on the impact of adjuvant radiotherapy.

In favor of adjuvant radiotherapy	Against adjuvant radiotherapy
Oh et al. (31) 348 adults (337 GII); literature review: 5- and 10-year PFS: 98% and 98% vs. 65% and 50% vs. 45% and 30%—GTR vs. STR + RT vs. STR	Wang et al. (32) 636 adults (621 G II); SEER database: not reported OS/PFS with respect to RT
Rodriguez et al. (18) 2,408 pts (2,132 spine and brain G II); SEER database: not reported OS/PFS with respect to RT	Roldan Urgoiti et al. (15) 139 pts (106 brain and spine G II): not reported OS/PFS with respect to RT
Savoor et al. (33) 69 adults (42 G II): 10-year PFS and LC: 77% and 86% vs. 68% and 50%—SRT + RT vs. SRT	Tarapore et al. (34) 134 pts (101 G II): 10-year PFS: 88% vs. 70% vs. 82%—GTR vs. STR + RT vs. STR.
Gomez et al. (35) 37 pts (26 low G other than myxopapillary): not reported OS/PFS with respect to RT	Brown et al. (8) 1058 pts (1019 G II); NCD analysis: 5-year OS—95% vs. 95%—RT vs. no RT
Zou et al. (36) literature review of 13 children: not reported OS/PFS with respect to RT	Sun et al. (37) literature review of 138 pts: not reported OS/PFS with respect to RT
Lin et al. (38) 20 pts (13 G II): 10-year LC and OS: 100% and 100%—GTR and STR + RT	Lin et al. (39) 64 children; SEER database: 10y OS: 87% vs. 75%—RT vs. no RT
Lee et al. (45) 19 pts: 5-year LC and OS: 100% and 100%—GTR and STR + RT	Lee et al. (40) 88 pts (61 G II): 10-year DFS: 55% vs. 90%—RT vs. no RT (whole group)
	Wang et al. (41) literature review of 169 pts: 5-year PFS: 85% vs. 95%—RT vs. no RT
	Vera-Bolanos et al. (43) 282 adults (212 spine and brain G II): 10-year PFS: 75% vs. 58%—GTR vs. GTR + RT; 10-year PFS: 58% vs. 50%—STR vs. STR+RT
	Amirian et al. (46) 367 pts (348 GII spine and brain); SEER analysis: not reported OS/PFS with respect to RT

On the left studies in favor of adjuvant radiotherapy, on the right against postoperative irradiation (7, 15, 18, 31–41, 43, 45, 46).

DFS, disease-free survival; G, grade; GTR, gross total resection; LC, local control; OS, overall survival; PFS, progression-free survival; pts, patients; RT, radiotherapy; SEER, the Surveillance, Epidemiology and End Results; STR, subtotal resection; vs., versus.

Based on the best available and most recent data, the NCCN and EANO guidelines recommend adjuvant RT following STR of G2E (2, 3). Several reports showed that adjuvant irradiation could significantly improve local control, especially in patients where GTR was not performed (31, 33, 35, 36, 45). Authors concluded that although adjuvant RT may not ultimately affect OS, decreasing recurrence can appreciably benefit the patient outcomes by avoiding repeated surgeries, which are associated with significant morbidities (31, 33). RT is typically targeted to the spinal level of disease (GTV) with a margin accounting for microscopic spread (CTV) and intra- and infrafraction motion (PTV). The craniospinal irradiation is reserved for cases with dissemination diagnosed based on MR or cerebrospinal fluid analysis with possible boost on gross visible disease. Taking into account the tolerance dose of 45–50 Gy for spinal cord tissues and the studies which did not show an improvement in the outcome after the application of higher doses, the irradiation to the total dose of 45–50.4 Gy with a fraction dose of 1.5–1.8 Gy is recommended (higher doses are used in case of tumors in the spinal canal in regions of the nerve roots and the cauda equina below the spinal cord) as providing satisfactory results (2, 3, 31, 33, 35, 38, 47, 49, 50). In contrast to that findings but in accordance with other researchers, we did not find adjuvant RT as influencing survival (8, 32, 34, 37, 40, 46). However, it is worth mentioning that vast majority of our patients, similarly to the study by Lin et al., received RT even in case of GTR, so it is hard to advise not to irradiate (39). This also reflects how hard is the clinical decision-making process of selecting patients suitable for radiation, which makes one of the drawbacks of retrospective studies since the goal is not to verify the best solution but to cure the patient. Similar to our results were observed by Gomez et al., and they still advise to administer adjuvant RT in case of STR or biopsy (35). Sun et al. in their literature review included cases in which adjuvant RT was used only in 27% of patients and authors concluded that adjuvant treatment did not prevent from tumor recurrence and those who received irradiation had worse PFS. It is worth mentioning that indication for adjuvant treatment were not analyzed with regard to the EOR in that study (37). Lee et al. did not report the outcome with regard to the tumor grade which could have influenced the observed differences (40). What is more, among studies against RT, there are four which analyzed SEER or NCDB data. As shown in the study by Metellus et al. in which over 40% cases were misclassified, national database evaluations could raise concerns about proper histopathological diagnosis (8, 16, 32, 39, 46). What is important, while the use of RT remains controversial, the modern planning techniques have increased precision of targeting, decreasing the effects on surrounding tissue and the risk of possible complications. Because recurrences can occur many years after the treatment, regardless of the EOR, long-term follow-up with MR is recommended. Older age, male sex, other than classical G2 pathological types (cellular, papillary, giant cell types) were found as negatively influencing the outcome (8, 32, 37, 39, 41).

### Study Limitations

There are several limitations of our study. Foremost, the retrospective nature of this study hampers the value of our findings. The other is the long duration of time during which patients were eligible for inclusion and lack of molecular testing which is now a pathologic standard in case of neurooncologic lesions. What is more, we did not perform the review of the pathologic diagnosis for the purpose of this study, but since our cancer center lacks neurosurgery department and patients had surgery in many different hospitals all over the region, we were not able to collect the tissue samples and evaluate them due to logistic reasons. As the study period covered very long time—from 1985 to 2019, some of those specimens are no longer available, because according to the Polish medical law, tissues are stored in pathology department for a period of 20 years and then disposed. However, the analysis of pathology department which conducted the pathologic evaluation showed that over 70% had confirmation of very reliable diagnosis. What is more, our findings are strengthened by the relatively large number of cases included, long follow-up, and number of information regarding treatment applied. Last but not least, the general consensus regarding the management of ependymomas has evolved based on the institutional experience and retrospective studies like ours. However, taking into account changes in pathological classification and introduction of molecular subgroups, there is a need for multi-institutional or cooperative group prospective studies or registries to better define these populations and inform future clinical investigations.

## Conclusions

Radiotherapy applied in the case of patients with G2 ependymoma is a valuable treatment of recurrent disease. Patients with brain lesions after nonradical surgery might benefit from local irradiation in terms of progression-free survival. The low number of events in patients with spinal tumors does not allow for an unequivocal conclusion regarding the role of RT. Also, the role of irradiation after radical resection remains debatable. RT as part of the treatment of recurrence has positive impact on overall survival.

## Data Availability Statement

The original contributions presented in the study are included in the article/supplementary material. Further inquiries can be directed to the corresponding author.

## Ethics Statement

The studies involving human participants were reviewed and approved by The Institutional Review Board committee in MSC National Research Institute of Oncology in Gliwice (number: KB/430-05/20). Written informed consent for participation was not required for this study in accordance with the national legislation and the institutional requirements.

## Author Contributions

Conceptualization: AN. Methodology: AN. Software: AN. Validation: AN. Formal analysis: AN. Investigation: AN. Resources: AN. Data curation: AN. Writing—original draft preparation: AN and LM. Writing—review and editing: AN, LM, and WM. Visualization: AN. Supervision: LM and WM. Project administration: AN. All authors have read and agreed to the published version of the manuscript.

## Conflict of Interest

The authors declare that the research was conducted in the absence of any commercial or financial relationships that could be construed as a potential conflict of interest.

## Publisher’s Note

All claims expressed in this article are solely those of the authors and do not necessarily represent those of their affiliated organizations, or those of the publisher, the editors and the reviewers. Any product that may be evaluated in this article, or claim that may be made by its manufacturer, is not guaranteed or endorsed by the publisher.
